# The bias of experimental design, including strain background, in the determination of critical *Streptococcus suis* serotype 2 virulence factors

**DOI:** 10.1371/journal.pone.0181920

**Published:** 2017-07-28

**Authors:** Jean-Philippe Auger, Sarah Chuzeville, David Roy, Annabelle Mathieu-Denoncourt, Jianguo Xu, Daniel Grenier, Marcelo Gottschalk

**Affiliations:** 1 Swine and Poultry Infectious Diseases Research Center (CRIPA), Faculty of Veterinary Medicine, University of Montreal, Saint-Hyacinthe, Quebec, Canada; 2 Research Group on Infectious Diseases in Production Animals (GREMIP), Faculty of Veterinary Medicine, University of Montreal, Saint-Hyacinthe, Quebec, Canada; 3 Collaborative Innovation Center for Diagnosis and Treatment of Infectious Diseases, National Institute for Communicable Disease Control and Prevention, Chinese Center for Disease Control and Prevention, Beijing, China; 4 Oral Ecology Research Group (GREB), Faculty of Dentistry, Laval University, Quebec City, Quebec, Canada; Oregon Health & Science University, UNITED STATES

## Abstract

*Streptococcus suis* serotype 2 is an important porcine bacterial pathogen and emerging zoonotic agent mainly responsible for sudden death, septic shock, and meningitis. However, serotype 2 strains are genotypically and phenotypically heterogeneous. Though a multitude of virulence factors have been described for *S*. *suis* serotype 2, the lack of a clear definition regarding which ones are truly “critical” has created inconsistencies that have only recently been highlighted. Herein, the involvement of two factors previously described as being critical for *S*. *suis* serotype 2 virulence, whether the dipeptidyl peptidase IV and autolysin, were evaluated with regards to different ascribed functions using prototype strains belonging to important sequence types. Results demonstrate a lack of reproducibility with previously published data. In fact, the role of the dipeptidyl peptidase IV and autolysin as critical virulence factors could not be confirmed. Though certain *in vitro* functions may be ascribed to these factors, their roles are not unique for *S*. *suis*, probably due to compensation by other factors. As such, variations and discrepancies in experimental design, including *in vitro* assays, cell lines, and animal models, are an important source of differences between results. Moreover, the use of different sequence types in this study demonstrates that the role attributed to a virulence factor may vary according to the *S*. *suis* serotype 2 strain background. Consequently, it is necessary to establish standard experimental designs according to the experiment and purpose in order to facilitate comparison between laboratories. Alongside, studies should include strains of diverse origins in order to prevent erroneous and biased conclusions that could affect future studies.

## Introduction

*Streptococcus suis* is an important porcine bacterial pathogen and emerging zoonotic agent mainly responsible for sudden death (pigs), septic shock (humans), and meningitis (both species) [1]. Of the different described serotypes based on the presence of the capsular polysaccharide or its respective genes, serotype 2 is regarded as not only the most widespread worldwide, but also the most virulent, responsible for the majority of porcine and human cases of *S*. *suis* infection [2]. Using multilocus sequence typing, the distribution of the most important sequence types (STs) of *S*. *suis* serotype 2 has been determined worldwide [2]. Moreover, recent studies have evaluated the virulence of these important STs using well-characterized mouse models of infection, where virulence is defined based on the capacity of a strain to induce clinical disease and mortality [3, 4]. The ST7 strain responsible for the human outbreaks of 1998 and 2005 in China [5] is highly virulent whereas European ST1 strains are virulent; on the other hand, ST25 strains, typically recovered in North America, are of intermediate virulence [3].

Over the years, a multitude of virulence factors, presently totaling more than 150, have been described to be implicated in the *S*. *suis* serotype 2 pathogenesis in pigs and humans [6–9]. However, the lack of a clear definition regarding what constitutes a virulence factor for *S*. *suis*, which generally differs from one laboratory to another, and the fact that many of these have redundant roles, have greatly hindered the identification of truly “critical” virulence factors and created inconsistencies throughout the literature [9]. Indeed, of the different factors described so far, at least 76 have been reported to be implicated in virulence, while 35 of these were critical for virulence since their absence resulted in avirulence [9]. Alongside, many putative virulence factors are present in certain virulent strains but not in others, such as the suilysin, muramidase-released protein (MRP), and extracellular protein factor, which currently serve as virulence markers for Eurasian *S*. *suis* serotype 2 strains only, since these are often absent in North American strains [2, 9, 10]. In fact, the North American strains that do possess the MRP are associated with lower virulence [4, 10]. Moreover, the important roles played by a putative virulence factor might depend on the genetic background of the selected strain. Finally, the use of differing experimental designs, including *in vitro* assays, cell lines, and animal models, have made it extremely difficult to accurately compare results between laboratories [9].

Herein, the involvement of two *S*. *suis* serotype 2 virulence factors previously described as being critical were evaluated with regards to different ascribed functions using prototype strains belonging to three of the most important STs (ST1, ST7, and ST25). These proteins, which served as tools, were chosen among the more than 150 putative virulence factors described for *S*. *suis* on the basis of being present in strains from these three backgrounds. The dipeptidyl peptidase IV (DPPIV), originally studied using a ST7 isolate recovered from a human case during the 2005 Chinese outbreak [11], is a serine protease widely distributed in eukaryotes and bacteria that has been suggested to contribute to bacterial pathogenesis [12]. Meanwhile, the autolysin [13], originally studied using a ST378 strain recovered from a diseased pig in China [14], is a peptidoglycan hydrolase implicated in various bacterial functions such as cell wall turnover, cell division, and cell separation [13]. Consequently, the aim of this study was to evaluate the bias of experimental design, including strain background, in the determination of *S*. *suis* serotype 2 virulence factors in order to better clarify the recently highlighted controversy caused by inconsistencies plaguing this field of research.

## Materials and methods

### Ethics statement

This study was carried out in accordance with the recommendations of the guidelines and policies of the Canadian Council on Animal Care and the principles set forth in the Guide for the Care and Use of Laboratory Animals. The protocols and procedures were approved by the Animal Welfare Committee of the University of Montreal (Permit Number: Rech-1570).

### Bacterial strains and growth conditions

The three well-characterized and highly encapsulated intermediate to highly virulent prototype wild-type *S*. *suis* serotype 2 strains and their isogenic mutants used in this study are listed in Table 1. Strains were minimally passaged and virulence routinely tested using cell-based assays and experimental infection models. The *S*. *suis* strains were cultured in Todd Hewitt broth (THB; Becton Dickinson, Mississauga, ON, Canada). For adhesion assays, bacterial cultures were prepared as previously described [15]. Briefly, upon reaching the mid-exponential phase, bacteria were washed twice with phosphate-buffered saline (PBS), pH 7.3, and resuspended in PBS for adhesion to fibronectin or cell culture medium (Gibco, Burlington, ON, Canada) for adhesion to porcine epithelial cells (described below). For experimental infections, early stationary phase bacteria were washed twice in PBS and resuspended in THB [4, 16, 17]. Bacterial cultures were appropriately diluted and plated on THB agar to accurately determine bacterial concentrations. mRNA expression of the *dppIV* and *atl* genes was determined to be similar between the three wild-type strains under the growth conditions used in this study as quantified by RT-qPCR (data not shown). The *Escherichia coli* strain and different plasmids used in this study are also listed in Table 1. When needed, antibiotics (Sigma, Oakville, ON, Canada) were added to the media at the following concentrations: for *S*. *suis*, spectinomycin at 100 μg/mL and chloramphenicol at 5 μg/mL; for *E*. *coli*, kanamycin and spectinomycin at 50 μg/mL and chloramphenicol at 30 μg/mL.

**Table 1:** Strains and plasmids used in this study.

Strains/plasmids	General characteristics	Reference
***Streptococcus suis***		
P1/7	Wild-type, virulent European ST1 strain isolated from a case of pig meningitis in the United Kingdom	[18]
P1/7Δ*dppIV*	Isogenic mutant derived from P1/7; in frame deletion of the *dppIV* gene	This study
P1/7Δ*atl*	Isogenic mutant derived from P1/7; in frame deletion of the *atl* gene	This study
SC84	Wild-type, highly virulent ST7 strain isolated from a case of human streptococcal toxic shock-like syndrome during the 2005 outbreak in China	[19]
SC84Δ*dppIV*	Isogenic mutant derived from SC84; in frame deletion of the *dppIV* gene	This study
SC84Δ*atl*	Isogenic mutant derived from SC84; in frame deletion of the *atl* gene	This study
89–1591	Wild-type, intermediate virulent North American ST25 strain isolated from a case of pig sepsis in Canada	[20]
89–1591Δ*dppIV*	Isogenic mutant derived from 89–1591; in frame deletion of the *dppIV* gene	This study
89–1591Δ*atl*	Isogenic mutant derived from 89–1591; in frame deletion of the *atl* gene	This study
***Escherichia coli***		
TOP 10	F^-^ mrcA Δ(mrr-hsdRMS-mcrBC) φ80 lacZΔM15 ΔlacX74 recA1 araD139 Δ(ara-leu) 7697 galU galK rpsL (Str^R^) endA1 nupG	Invitrogen
**Plasmids**		
pCR2.1	Ap^r^, Km^r^, oriR(f1) MCS oriR (ColE1)	Invitrogen
pSET-4s	Thermosensitive vector for allelic replacement in *S*. *suis*. Replication functions of pG + host3, MCS oriR pUC19 lacZ Sp^R^	[21]
p4Δ*dppIV*	pSET-4s carrying the construct for *dppIV* allelic replacement	This work
p4Δ*atl*	pSET-4s carrying the construct for *atl* allelic replacement	This work

### DNA manipulations

*S*. *suis* genomic DNA was extracted using the InstaGene Matrix solution (BioRad Laboratories, Hercules, CA, USA). Mini-preparations of recombinant plasmids were carried out using the QIAprep Spin Miniprep Kit (Qiagen, Valencia, CA, USA). Restriction enzymes and DNA-modifying enzymes (Fisher Scientific, Ottawa, ON, Canada) were used according to the manufacturer’s recommendations. Oligonucleotide primers (Table 2) were obtained from Integrated DNA Technologies (Coralville, IA, USA) and PCRs carried out with the iProof proofreading DNA polymerase (BioRad Laboratories, Mississauga, ON, Canada) or with the Taq DNA polymerase (Qiagen). Amplification products were purified using the QIAquick PCR Purification Kit (Qiagen) and sequenced using an ABI 310 Automated DNA Sequencer and the ABI PRISM Dye Terminator Cycle Sequencing Kit (Applied Biosystems, Carlsbad, CA, USA).

**Table 2:** Oligonucleotide primers used for the construction of the *S*. *suis* dipeptidyl peptidase IV (*dppIV*) and autolysin (*atl*) mutants used in this study.

Name	Primer Sequence (5’-3’)
ST1 & ST7Δ*dppIV*_1	GATCCAGCTCCAACTCCAATTC
ST1 & ST7Δ*dppIV*_2	TTGGGATCATGCACCACACC
ST1 & ST7Δ*dppIV*_3	CCCCCGGGGAAGTTCCGGCACCAATTCCAG
ST1 & ST7Δ*dppIV*_4	TCCGTCTACTTGCAAAATTCTCAATGGCAAATCCAC CTTG
ST1 & ST7Δ*dppIV*_5	TTGCCATTGAGAATTTTGCAAGTAGACGGAGGTC
ST1 & ST7Δ*dppIV*_6	CGGGATCCGTTCGGAACATACCAAAGGG
ST25Δ*dppIV*_1	CAATAAGAAGCCCAGCAAGAG
ST25Δ*dppIV*_2	GTTGCAAGTACCCTCATTTCC
ST25Δ*dppIV*_3	TCGCTTCCTTAAGCTGGTC
ST25Δ*dppIV*_4	TCCGTCTACTTGCAAAATTCTCAATGGCAAATC CACCTTG
ST25Δ*dppIV*_5	TTGCCATTGAGAATTTTGCAAGTAGACGGAGGTC
ST25Δ*dppIV*_6	GCCACTTGGTCAGACAAAG
ST1 & ST7Δ*atl*_1	CCAGTTGTAGCAGCAGAG
ST1 & ST7Δ*atl*_2	ACCAGCATGAAAAGAACAGATG
ST1 & ST7Δ*atl*_3	CATTTAACTGATGATGAAAAAG
ST1 & ST7Δ*atl*_4	ATACCAATTCATTACACCTTGCTCCTTTATGTATTTCACATGTAA
ST1 & ST7Δ*atl*_5	TTACATGTGAAATACATAAAGGAGCAAGGTGTAATGAATTGGTAT
ST1 & ST7Δ*atl*_6	GTACTTACAAAGAGCCAACAG
ST25Δ*atl*_1	GGAAGTGCTACACTACCGTC
ST25Δ*atl*_2	GACCAGCATGAAAAGAAC
ST25Δ*atl*_3	CGGAGCTGTTCCAGTT
ST25Δ*atl*_4	CAAGGCGAGTGTGGTACTCCTTTATGTATTTCACATGTAA
ST25Δ*atl*_5	TTACATGTGAAATACATAAAGGAGTACCACACTCGCCTTG
ST25Δ*atl*_6	GCAGATTTAATTACTTTCTTTAGC

### Construction of the isogenic dipeptidyl peptidase IV and autolysin mutants

The DNA genome sequences of the wild-type *S*. *suis* strains were used. In-frame deletions of the *dppIV* or *atl* genes were constructed using splicing-by-overlap-extension PCRs as previously described [11, 13, 22]. Overlapping PCR products were cloned into pCR2.1 (Invitrogen, Burlington, ON, Canada), extracted with EcoRI, recloned into the thermosensitive *E*. *coli*–*S*. *suis* shuttle plasmid pSET4s, and digested with the same enzyme, giving rise to the knockout vector p4Δ*dppIV* or p4Δ*atl*. Electroporation of the three *S*. *suis* wild-type strains and procedures for isolation of the mutants were previously described [23]. Allelic replacement was confirmed by PCR and DNA sequencing analysis. Amplification products were purified with the QIAgen PCR Purification Kit (Qiagen) and sequenced as described above. mRNA expression of upstream and downstream genes flanking the *dppIV* and *atl* genes in the mutant strains was confirmed by RT-PCR, validating in-frame gene deletion (data not shown). Growth of the different mutant strains was similar to that of the wild-type strains (data not shown).

### *S*. *suis* adhesion to human fibronectin (microtiter plate binding assay)

Fibronectin adhesion assays were carried out as previously described [24]. Briefly, microtiter plates were coated with different concentrations of human plasma fibronectin (Sigma-Aldrich, St-Louis, MO, USA), ranging from 0 to 10 μg/mL, in 0.1 M carbonate buffer, pH 9.6. Formaldehyde-killed bacterial suspensions (equivalent to 1 x 10^8^ colony forming units [CFU]/mL) of the different wild-type and mutant strains were added and the plates incubated for 2 h at 37°C. It was previously demonstrated that killing of *S*. *suis* using 0.2% formaldehyde does not affect its capacity to bind fibronectin [24]. An anti-*S*. *suis* serotype 2 rabbit serum followed by a horseradish peroxidase-labelled anti-rabbit IgG conjugate (Jackson Immunoresearch Laboratories Inc., West Grove, PA, USA) were used. The anti-*S*. *suis* serum equally recognized the wild-type and mutant strains by enzyme-linked immunosorbent assay (ELISA) [24]. The enzyme substrate, 3,30,5,50-tetramethylbenzidine (Zymed, San Francisco, CA, USA) was used according to the manufacturer’s instructions, the reaction stopped using 1 N H_2_SO_4_, and the optical density measured at 450 nm using a microtiter plate reader (Molecular Devices, Menlo Park, CA, USA). Uncoated wells served as background controls. Casein-coated wells served as a control for non-specific adhesion of *S*. *suis* to protein-coated wells.

### *S*. *suis* adhesion to porcine tracheal epithelial cells

The newborn porcine tracheal epithelial cell line (NPTr) was used and cultured until confluent as previously described [25]. Cells were infected with *S*. *suis* (10^6^ CFU/well; multiplicity of infection [MOI] = 10) by removing the cell culture medium, adding 1 mL of bacteria in cell culture medium without antibiotics, and incubating for 2 h at 37°C with 5% CO_2_ as previously described [15]. Following incubation, cells were washed five times with PBS to remove non-adherent bacteria and lysed using 1 mL of sterile water. The lysates were appropriately diluted and plated on THB agar to quantify adhered bacteria. Alongside, the last wash was plated to confirm absence of non-adhered bacteria. The percentage of adhered bacteria was calculated according to the following: CFU recovered 2 h post-incubation / inoculum x 100% [11, 13].

### *S*. *suis* biofilm formation capacity

The biofilm formation capacity of the different wild-type and mutant strains was determined as previously described [26]. Moreover, the protocol used was identical to that described by Ju *et al*., including the use of 2 mg/mL of porcine fibrinogen (Sigma-Aldrich), incubation for 24 h at 37°C, subsequent staining with crystal violet, and measurement of the optical density at 575 nm [13].

### *S*. *suis in vivo* virulence mouse infections

A well-standardized C57BL/6 mouse model of infection was used [3, 4, 17]. These studies were carried out in strict accordance with the recommendations of and approved by the University of Montreal Animal Welfare Committee guidelines and policies, including euthanasia to minimize animal suffering through the use of humane endpoints, applied throughout this study when animals were seriously affected since mortality was not an endpoint measurement. No additional considerations or housing conditions were required. All staff members received the required animal handling training as administered by the University of Montreal Animal Welfare Committee. A total of 140 six-week-old male and female C57BL/6 mice (Jackson Research Laboratories, Bar Harbour, MA, USA) were used (10 to 15 mice/group) in this study. Mice were inoculated with 5 x 10^7^ CFU via the intraperitoneal route and health and behavior monitored at least thrice daily until 72 h post-infection (p.i.) and twice thereafter until the end of the experiment (14 days p.i.) for the development of clinical signs of sepsis, such as depression, swollen eyes, rough hair coat, and lethargy. Mice were also monitored for the development of clinical signs of meningitis. Clinical scores were determined according to the grid approved by the University of Montreal Animal Welfare Committee (S1 Appendix) and required actions undertaken. Mice were immediately euthanized upon reaching endpoint criteria using CO_2_ followed by cervical dislocation. No mice died before meeting endpoint criteria and all surviving mice were euthanized as described above at the end of the experiment (14 days p.i.). Blood samples were collected from the caudal vein of surviving mice 24 h p.i. and plated as previously described [4].

### Statistical analyses

Significant differences were determined using the t-test, Mann-Whitney Rank sum test, one way ANOVA, and ANOVA on ranks, where appropriate. For *in vivo* virulence experiments, survival was analyzed using the LogRank test. A *p* < 0.05 was considered statistically significant.

## Results

### The *S*. *suis* serotype 2 dipeptidyl peptidase IV and autolysin are not major fibronectin-binding adhesins, regardless of the sequence type of the strain used

**Fig 1:**
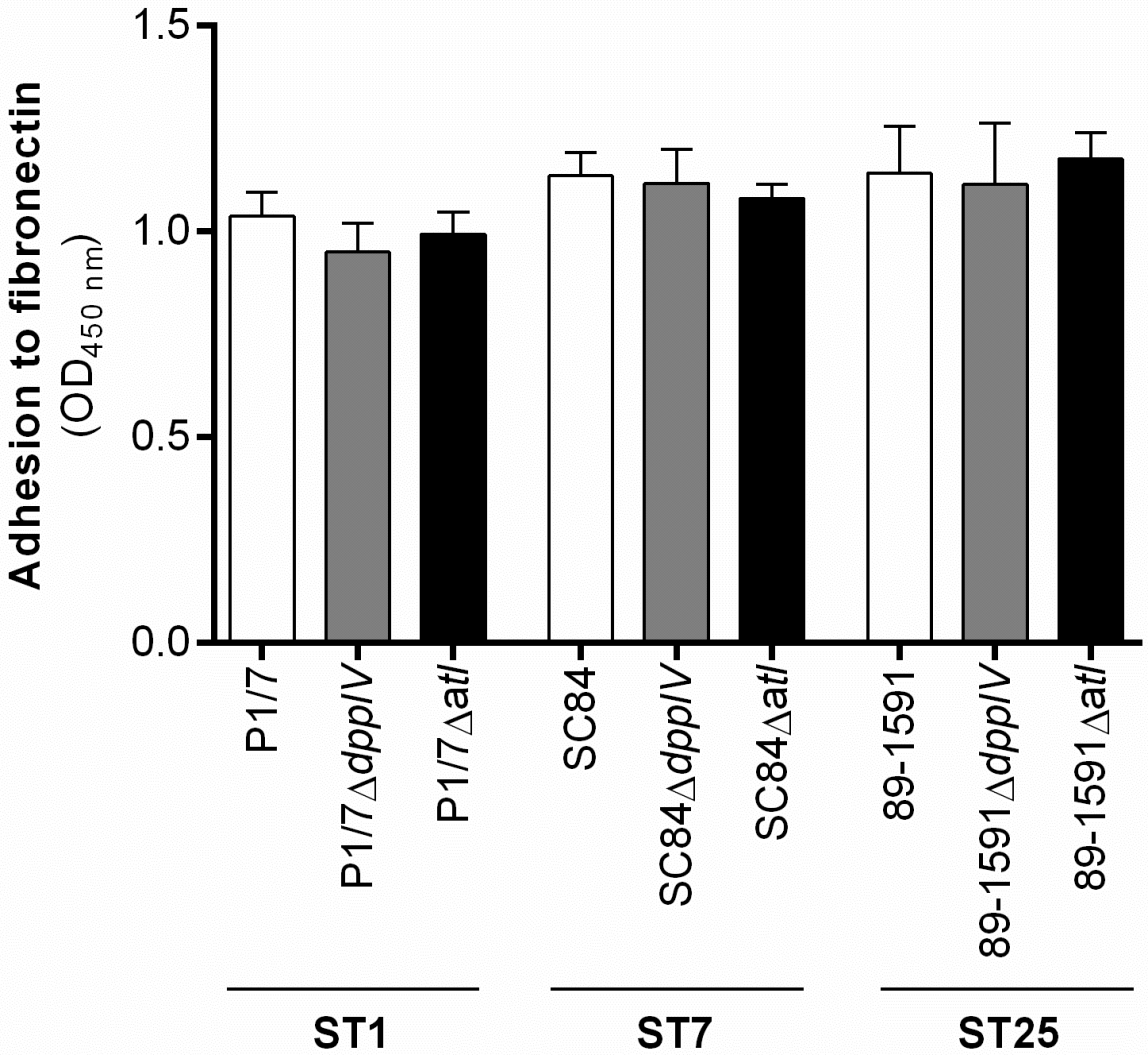
The *S*. *suis* serotype 2 dipeptidyl peptidase IV and autolysin are not involved in adhesion to fibronectin, regardless of the sequence type (ST) of the strain used. Adhesion of different wild-type strains and dipeptidyl peptidase IV (DPPIV)- or autolysin (Atl)-deficient mutants to human plasma fibronectin (10 μg/mL), as determined by ELISA after 2 h of incubation. The optical density (OD) was measured at 450 nm and values corrected using the appropriate controls. Results are expressed as mean ± SEM obtained from three independent experiments.

Adhesion to host extracellular matrix (ECM) components is an important and often crucial initial step of the bacterial pathogenesis [8]. Amongst the different components of the ECM is plasma fibronectin, to which both the DPPIV of a ST7 strain and the autolysin of a ST378 strain were previously reported to bind [11, 13]. Results showed similar levels of adhesion to fibronectin between the wild-type ST1, ST7, and ST25 strains, as measured by ELISA (Fig 1). The role of the DPPIV and autolysin in binding human fibronectin was then evaluated using their respective isogenic mutants. In the presence of 10 μg/mL of fibronectin (concentration shown to be optimal for *S*. *suis*; data not shown), no significant differences were observed between the adhesion of the Δ*dppIV* or Δ*alt* mutants and their respective wild-type strains, regardless of the ST of the strain used (Fig 1). Similar results were obtained using lower concentrations of fibronectin (data not shown). These results suggest that the DPPIV and autolysin are not major human fibronectin-binding adhesins for *S*. *suis* serotype 2.

### The *S*. *suis* serotype 2 dipeptidyl peptidase IV, unlike the autolysin, does not play a major role in adhesion to porcine tracheal epithelial cells

**Fig 2:**
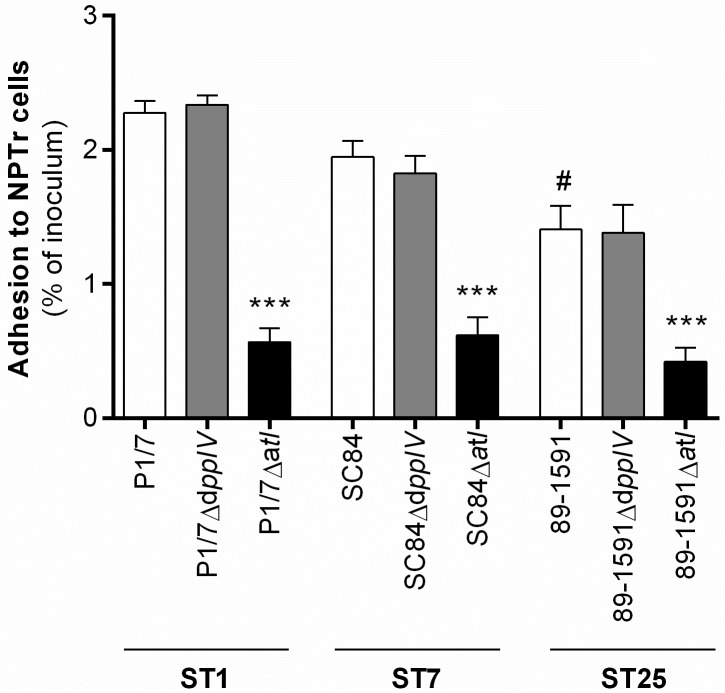
The *S*. *suis* serotype 2 dipeptidyl peptidase IV is not involved in adhesion to porcine tracheal epithelial cells, regardless of the sequence type (ST) of the strain used, unlike the autolysin. Adhesion of different wild-type strains and dipeptidyl peptidase IV (DPPIV)- or autolysin (Atl)-deficient mutants to porcine epithelial cells was evaluated after 2 h of incubation with bacteria (MOI = 10). Results are expressed as mean ± SEM obtained from three independent experiments and represent the percentage of adhered inoculum. # indicates a significant difference (*p* < 0.01) between the wild-type ST1 strain P1/7 and ST25 strain 89–1591; *** (*p* < 0.001) between the wild-type strain and its Atl-deficient mutant.

Adhesion to host cells is a requirement for subsequent interactions, including cell activation and establishment of the disease [8]. Indeed, it was previously suggested that both the DPPIV [11] and autolysin [13] are implicated in adhesion of *S*. *suis* serotype 2 to the human laryngeal epithelial cell line HEp-2. Herein, adhesion of the different wild-type strains and the role of the DPPIV and autolysin in adhesion to the porcine tracheal epithelial cell line NPTr was determined after 2 h of incubation with a MOI = 10 and results expressed as percentage of adhered inoculum. Adhesion of the wild-type ST1 strain was greatest, with that of the ST7 strain being intermediate, while the ST25 strain adhered the least, adhesion of which was significantly lower than that of the wild-type ST1 strain only (*p* < 0.01) (Fig 2). Moreover, results showed that the autolysin, but not the DPPIV, plays an important role in the adhesion to NPTr for the three wild-type strains (*p* < 0.001) (Fig 2). In fact, adhesion of the autolysin-deficient mutants was only 0.6%, 0.6%, and 0.4% of the inoculum for the ST1, ST7, and ST25 strains, respectively, in comparison to 2.3%, 1.9%, and 1.4% for their respective wild-type strains. As such, adherence of the three mutant strains was reduced by more than 70%. The DPPIV mutants, however, showed no differences compared to their respective wild-type strains regarding adhesion to porcine epithelial cells.

### The dipeptidyl peptidase IV is not involved in *S*. *suis* serotype 2 biofilm formation, while the implication of the autolysin is strain-dependent

**Fig 3:**
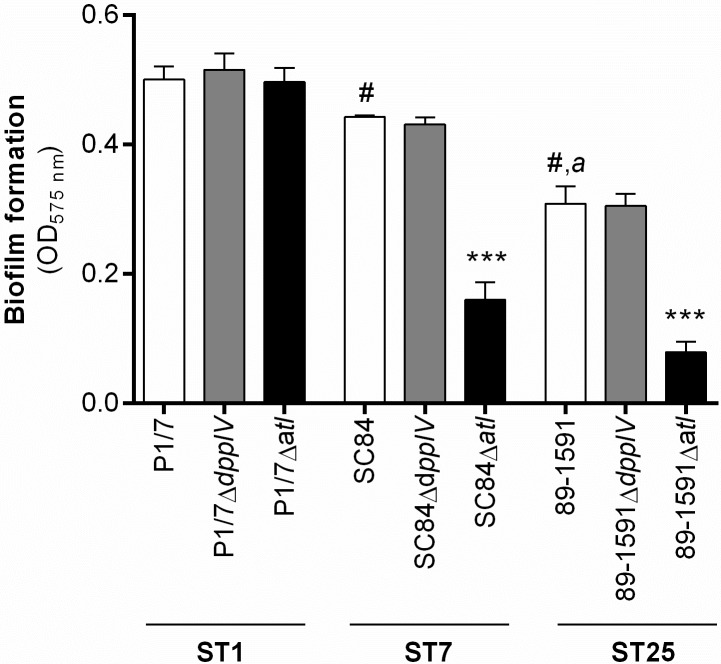
The dipeptidyl peptidase IV is not involved in *S*. *suis* serotype 2 biofilm formation, while implication of the autolysin is dependent on the sequence type (ST) of the strain used. Biofilm formation of different wild-type strains and dipeptidyl peptidase IV (DPPIV)- or autolysin (Atl)-deficient mutants in the presence of 2 mg/mL of porcine fibrinogen was evaluated after 24 h of incubation. The optical density (OD) was measured at 575 nm and values corrected using the appropriate controls. Results are expressed as mean ± SEM obtained from three independent experiments. # indicates a significant difference (*p* < 0.05) between the wild-type ST1 strain P1/7 and ST7 strain SC84 or ST25 strain 89–1591; *a* (*p* < 0.01) between the wild-type ST7 strain SC84 and ST25 strain 89–1591; *** (*p* < 0.001) between the wild-type strain and its Atl-deficient mutant.

Alongside adhesion to host ECM and cells, the capacity to form biofilm has been described as important for the *S*. *suis* pathogenesis, being involved in survival and propagation [8]. In order to enhance the biofilm formation capacity of the wild-type and mutant strains, culture medium was supplemented with porcine fibrinogen as previously described [26]. Results demonstrated that the wild-type ST1 strain produced significantly more biofilm than both the wild-type ST7 (*p* < 0.05) and ST25 strains (*p* < 0.01), though the ST7 strain produced more than the ST25 strain (*p* < 0.01) (Fig 3). While no data were available regarding a role of the DPPIV in biofilm formation by *S*. *suis*, the autolysin was previously reported to be implicated using a *S*. *suis* serotype 2 ST378 strain [13]. Using the same experimental design as previously used for the autolysin, the involvement of these two putative virulence factors in the capacity of the three wild-type *S suis* strains to form biofilm was evaluated. While the DPPIV was not involved in biofilm formation, regardless of the sequence type of the strain used, the autolysin of the ST7 and ST25 strains participated in biofilm formation (*p* < 0.001), but not that of the ST1 strain (Fig 3).

### The *S*. *suis* serotype 2 dipeptidyl peptidase IV and autolysin do not behave as critical virulence factors in an experimental model of *S*. *suis* serotype 2 infection

**Fig 4:**
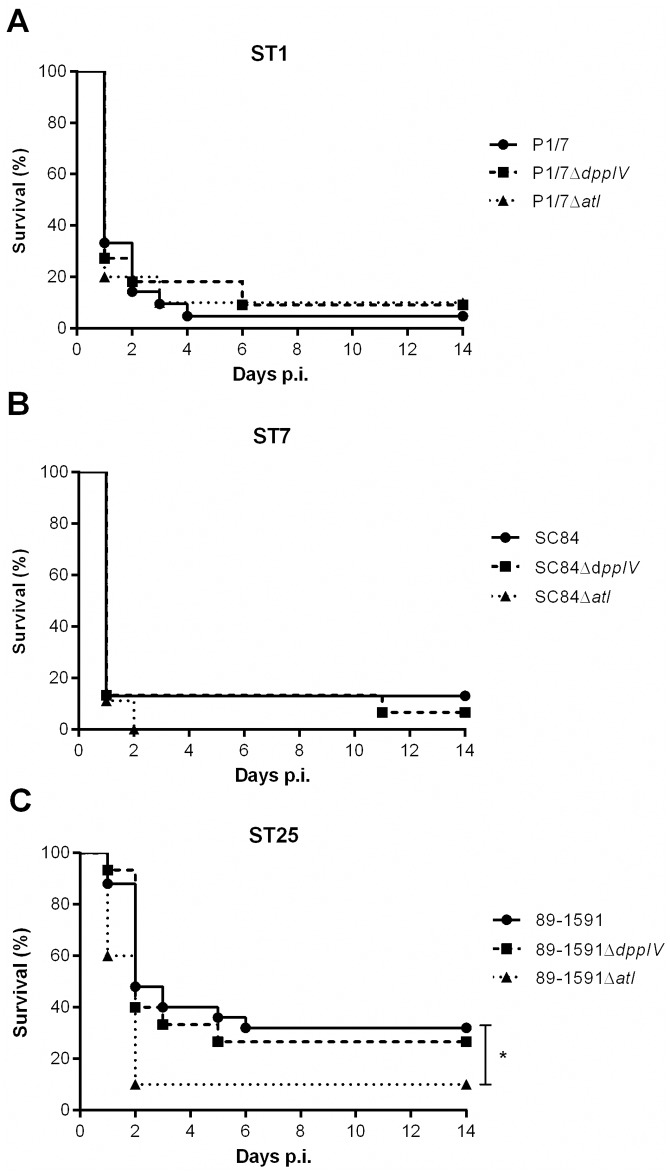
The *S*. *suis* serotype 2 dipeptidyl peptidase IV and autolysin are not implicated in host virulence in a C57BL/6 mouse model of infection, regardless of the sequence type (ST) of the strain, with the exception of a minor role for the autolysin of the ST25 strain. Survival of C57BL/6 mice infected with 5 x 10^7^ CFU of different wild-type and dipeptidyl peptidase IV (DPPIV)- or autolysin (Atl)-deficient mutants by intraperitoneal inoculation. (A) P1/7 (ST1) and its mutants, (B) SC84 (ST7) and its mutants, and (C) 89–1591 (ST25) and its mutants. * indicates a significant difference (*p* < 0.05) between the wild-type ST25 strain 89–1591 and its Atl-deficient mutant.

In order to evaluate the role of the DPPIV and autolysin in virulence, a well-characterized C57BL/6 mouse model of infection was used [4, 17]. In this model, mice succumb to septic shock during the systemic infection, after which surviving mouse are susceptible of developing meningitis. While no differences in survival were observed between mice infected with the wild-type ST1 (Fig 4A) and ST7 (Fig 4B) strains, results showed that the ST7 strain induced host death more rapidly than the ST1 strain. Moreover, the wild-type ST1 and ST7 strains caused significantly more mortality than the wild-type ST25 strain (Fig 4C) (*p* < 0.05): mortality caused by the ST25 strain was delayed and, unlike with the two other wild-type strains, partially due to the development of meningitis. For the virulent European ST1 strain (Fig 4A) and the highly virulent Chinese ST7 strain (Fig 4B), no significant role of the DPPIV and autolysin as critical virulence factors was observed. Moreover, this was also the case for the DPPIV of the intermediate virulent North American ST25 strain (Fig 4C). Meanwhile, and surprisingly, the ST25 autolysin-deficient mutant caused significantly higher mortality (*p* < 0.05) than its wild-type strain (Fig 4C). These results were confirmed in a subsequent infection (data not shown). Given these results, the *S*. *suis* serotype 2 DPPIV and autolysin are not significantly involved in virulence using a C57BL/6 mouse model of infection.

### The *S*. *suis* serotype 2 ST25 autolysin hinders bacterial survival in blood

**Fig 5:**
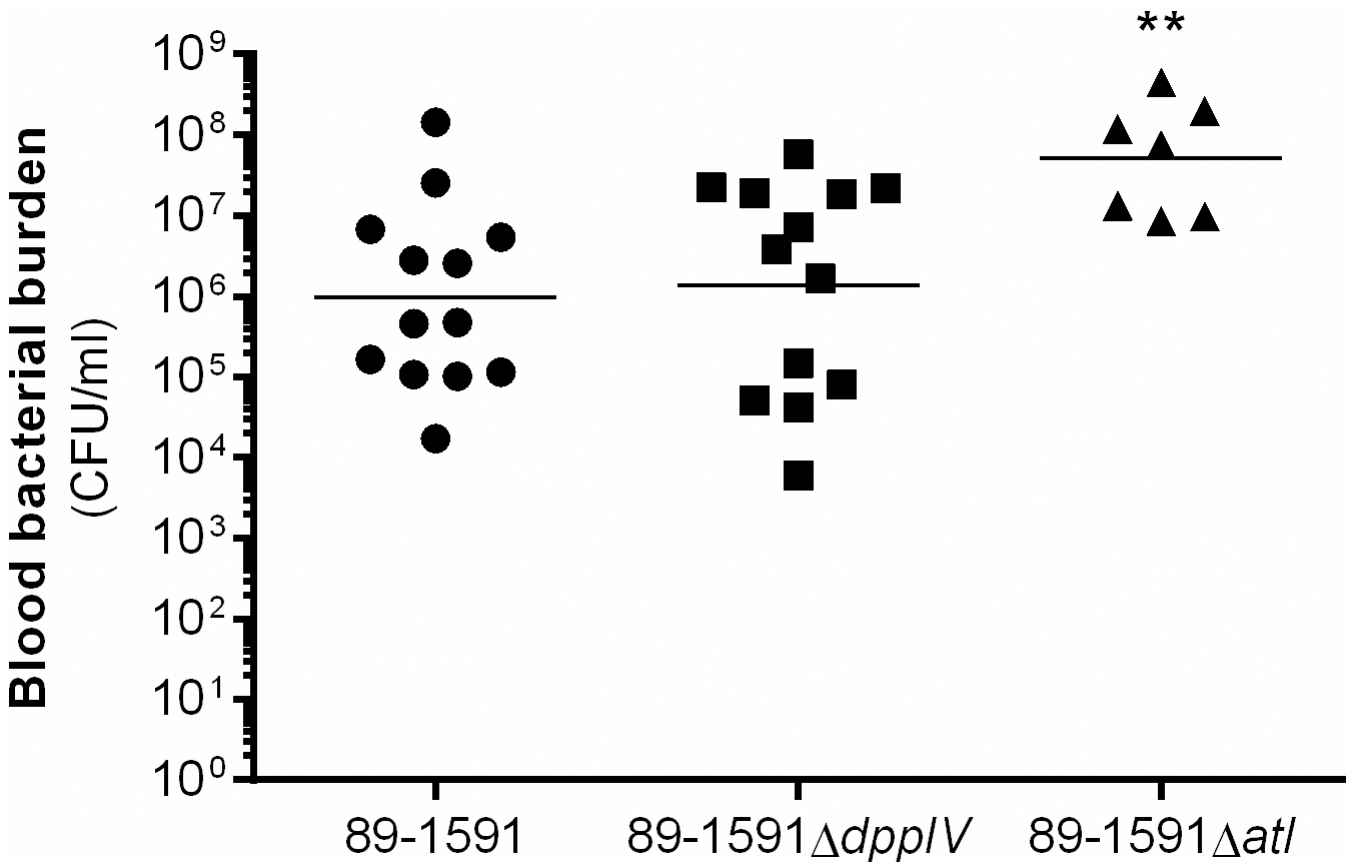
The autolysin of the ST25 strain, but not the dipeptidyl peptidase IV, hinders bacterial survival in the blood. Blood bacterial burden of surviving C57BL/6 mice 24 h following intraperitoneal inoculation of 5 x 10^7^ CFU of the ST25 strain 89–1591 and its dipeptidyl peptidase IV (DPPIV)- or autolysin (Atl)-deficient mutants. Results are expressed as geometric mean. ** indicates a significant difference (*p* < 0.01) between the wild-type ST25 strain 89–1591 and its Atl-deficient mutant.

Given the higher virulence of the autolysin-deficient ST25 strain, blood bacterial burden, which when uncontrolled may be responsible for *S*. *suis*-induced host death [4, 17], was evaluated 24 h p.i. for the wild-type ST25 strain and its two isogenic mutants. Indeed, blood bacterial burden was significantly higher (*p* < 0.01) in mice infected with the ST25 autolysin-deficient mutant than in those infected with either the wild-type strain or the DPPIV-deficient mutant, between which burdens were similar (Fig 5). This suggests that the autolysin might somewhat hinder survival of the ST25 strain in blood.

## Discussion

Though a multitude of virulence factors have been described for *S*. *suis* serotype 2, a clear definition of what constitutes a critical virulence factor is still lacking for this pathogen. This confusion has led to inconsistencies throughout the literature, resulting in a controversy that has only just been highlighted [9]. Consequently, the involvement of two *S*. *suis* serotype 2 factors, the DPPIV and autolysin, described as critical for virulence, was evaluated with regards to previously ascribed bacterial functions implicated in the *S*. *suis* pathogenesis using strains belonging to three important STs.

It is important to mention that the study by Ge *et al*. regarding the *S*. *suis* serotype 2 DPPIV was conducted using a ST7 strain (05ZYH33) isolated from the 2005 Chinese human outbreak [11]. Similarly, the ST7 strain used in the present study (SC84) was also isolated from a human case of streptococcal toxic shock-like syndrome during the same outbreak [19]. Since different isolates recovered from this outbreak have been reported to be highly similar [5], the influence of background should be minimal between these two strains. Meanwhile, the strain originally used to study the *S*. *suis* serotype 2 autolysin by Ju *et al*. is a ST378 (HA9801) according to the *S*. *suis* Multilocus Sequence Typing Website (http://ssuis.mlst.net) [13]. This ST was never reported beforehand and has not been reported since. To facilitate comparison of the methodologies used between the previous [11, 13] and present studies, the main experimental designs of the different assays are listed in Table 3. Since mRNA expression of *dppIV* and *atl* is similar between the three wild-type strains under the growth conditions used in this study, the differences observed herein are probably due to other variations between the strains, such as the presence/absence of putative virulence factors and/or differential expression of these factors.

**Table 3:** Comparison of the experimental design used in the previous and present studies to evaluate the role of the dipeptidyl peptidase IV and autolysin as virulence factors for *S*. *suis* serotype 2.

Experimental design	Previous study – Dipeptidyl peptidase IV [11]	Previous study – Autolysin [13]	This study
Sequence type (strain)	ST7 (05ZYH33)	ST378 (HA9801)	ST1 (P1/7) ST7 (SC84) ST25 (89–1591)
Adhesion to human fibronectin	Recombinant protein ELISA assay	Recombinant protein Western blot	Whole bacteria ELISA assay
Adhesion to epithelial cells	HEp-2 (Human laryngeal cells) MOI = 10 1 h or 2 h of incubation? % adhered inoculum	HEp-2 (Human laryngeal cells) MOI = 100 3 h of incubation % adhered inoculum? Adhesion of wild-type strain = 100%?	NPTr (Porcine tracheal cells) MOI = 10 2 h of incubation % adhered inoculum
Biofilm formation	Not evaluated	Microtiter plate assay after 24 h of incubation	Microtiter plate assay after 24 h of incubation
Virulence	Mouse Unknown (SPF), possibly BALB/c? Subcutaneous or intravenous? 1 x 10^8^ CFU/mouse?	Zebrafish Intraperitoneal 2 x 10^3^ to 2 x 10^7^ CFU/fish	Mouse C57BL/6 Intraperitoneal 5 x 10^7^ CFU/mouse

In order to evaluate the reproducibility of previously published results with strains from different backgrounds, the role of the *S*. *suis* serotype 2 DPPIV [11] and autolysin [13] in binding human plasma fibronectin was evaluated using a virulent European ST1 strain, the highly virulent clonal ST7 strain, and an intermediate virulent North American ST25 strain. The similar capacity of the three wild-type strains to bind human plasma fibronectin suggests this characteristic might be common to *S*. *suis* serotype 2, indicating a possibly universal role in the pathogenesis of this bacterium. Moreover, no significant implication of the *S*. *suis* serotype 2 DPPIV or autolysin in binding human fibronectin was observed when using isogenic mutants, regardless of the ST and fibronectin concentration. While these results cannot exclude those previously obtained using the recombinant DPPIV and autolysin as evaluated by ELISA and Western blot, respectively, they suggest that while the recombinant proteins themselves might bind human fibronectin, their absence is not sufficient to affect binding to this ECM component by *S*. *suis*. This lack of role when using isogenic mutants could be the result of compensation by one or more of the 18 factors currently known to bind fibronectin other than the DPPIV and autolysin: the fibronectin/fibrinogen-binding protein [27], enolase [28], Ssa (fibronectin-binding protein) [29], MRP [30], sortase A-anchored protein [31], catabolite control protein A [32], type II histidine triad protein [33], fructose-bisphosphate aldolase, lactate dehydrogenase, oligopeptide-binding protein OppA precursor, elongation factor Tu [34], sbp2 (putative pilin subunit) [35], translation elongation factor G, phosphoglycerate mutase, phosphoglycerate kinase, pyruvate dehydrogenase E1 component alpha subunit, and chaperonin GroEL [36]. Indeed, 29 different *S*. *suis* serotype 2 virulence factors have been described so far as binding ECM components [8, 9], which supports bacterial redundancy [37]. This redundancy was recently demonstrated for another putative virulence factor of *S*. *suis* serotype 2: the deletion of a single factor H-binding protein, of which ten have been described and another six have been proposed, is not sufficient to inhibit bacterial binding to factor H [38–41]. In fact, the simultaneous deletion of two of these genes, alongside a triple knockout for the capsular polysaccharide (also reported to bind factor H), remained insufficient to abolish binding to factor H, suggesting compensation by at least another bacterial factor, most probably one or more of these described proteins [38]. Consequently, a descriptive role for a bacterial protein alone is probably not sufficient to claim an important role in the pathogenesis of the infection, especially when other factors with redundant functions have already been described.

Adhesion to ECM components may subsequently led to interactions with host cells, which is an important step of bacterial pathogenesis [8]. It was previously demonstrated that the DPPIV of the ST7 strain and the autolysin of a ST378 strain are both implicated in adhesion to the human laryngeal epithelial cell line HEp-2 [11, 13]. Interestingly, the percentage of adhesion to epithelial cells obtained herein varied between STs, indicating a role of strain background. In fact, the ST1 and ST7 strains, which are virulent and highly virulent, respectively, adhered more than the intermediate virulent ST25 strain. Since adhesion to host cells may lead to cell invasion, differences in adhesion might influence host dissemination and virulence [6, 8]. Results obtained herein demonstrate that the DPPIV was not involved in adhesion to epithelial cells, regardless of the methodology used being similar (MOI and incubation time) and the ST being the same to that previously described [11]. However, certain differences in methodology still exist, such as the number of washes prior to cell lysis, the volume of water used to lyse the cells and, most notably, the origin of the cells used: human laryngeal epithelial cells versus porcine tracheal epithelial cells [11]. Though these two cell lines are both epithelial cells derived from the respiratory epithelium, it is impossible to ascertain that no other differences exist, such as histological differences between the trachea and larynx and the method used to immortalize the cells. Although it has been reported that adhesion to porcine and human epithelial cells by *S*. *suis* serotype 2 may be similar [15], the HEp-2 cells may not be an appropriate model for evaluating the role of all putative *S*. *suis* virulence factors since the respiratory route of infection has not been demonstrated for humans [9]. Consequently, these problems suggest that the experimental design used should be justified and the methodology standardized to ease comparison between studies and laboratories.

Meanwhile, a role of the autolysin in adhesion to host cells was confirmed when using porcine epithelial cells, and this for all three ST tested. Interestingly, it was previously reported that absence of the autolysin resulted in adhesion of only 50% of the inoculum by the mutant strain, while 100% of the wild-type strain inoculum adhered after 3 h of incubation [13]. These results greatly differ from those obtained in this study, in which approximately only 2% of the different wild-type strain inoculums and 0.5% of the autolysin-deficient mutant inoculums adhered to the epithelial cells. In fact, the high *S*. *suis* adhesion levels to epithelial cells reported by Ju *et al*. have never been observed by other researchers [13]. An hypothesis explaining the results of Ju *et al*. is that the elevated initial MOI (MOI = 100) and longer incubation time (3 h) may have led to bacterial replication within the wells [13]. Interestingly, despite differences in methodology and the origin of the cells, results obtained in this study arrived to the same conclusions for all three STs tested, suggesting that the role of this putative virulence factor in adhesion to epithelial cells might be universal for *S*. *suis* serotype 2.

Alongside adhesion to host ECM and cells, the capacity to form biofilm is an essential step of the bacterial pathogenesis involved in survival and propagation of the pathogen [8]. Interestingly, the three wild-type strains produced varying levels of biofilm, indicating a role of strain background concerning this capacity. These differences imply that choice of strain can have an important effect on the results obtained when evaluating certain characteristics or functions of *S*. *suis*. Moreover, the DPPIV was determined not to be involved in biofilm formation, regardless of the ST of the strain used. Though no role in biofilm formation had been attributed to this *S*. *suis* protein in the past, the lack of evaluation could have suggested otherwise, as exemplified by *Porphyromonas gingivalis*, for which the DPPIV is clearly involved in biofilm formation [42]. On the other hand, the autolysin was implicated in biofilm formation for the ST7 and ST25 strains, but not the ST1 strain. While the autolysin was previously reported to be implicated in biofilm formation by an ST378 strain, its absence resulted in a 25% decrease of production for the latter [13], while a decrease corresponding to nearly 70% of the biofilm formed by the wild-type ST7 and ST25 strains was observed herein. These results indicate a strain-dependent role of the *S*. *suis* serotype 2 autolysin with regards to this bacterial function. Consequently, these results demonstrate the impact of strain background and the bias introduced by this choice when evaluating virulence factors. This is important given that most studies regarding the evaluation of *S*. *suis* serotype 2 virulence factors have used ST1 or ST7 strains only.

When evaluating the implication of bacterial virulence factors, the ultimate demonstration remains the use of *in vivo* infection models. However, there exists a vast variety of *S*. *suis* serotype 2 animal infection models, which has complicated comparison of results. Of these the mouse is one of the most popular, with the inbred C57BL/6 breed being commonly used [3, 4, 17, 43]. Firstly, results obtained herein confirm previous studies in which the ST1 and ST7 strains were reported to both be virulent, with the ST7 strain inducing mortality more rapidly than the ST1 strain [3, 4]. Moreover, the wild-type ST25 strain caused less mortality and in a delayed time due to an important number of cases of meningitis, as previously reported [3, 4]. However, unlike previously reported for a ST7 strain, results obtained herein demonstrate that the DPPIV is not implicated in virulence and host death, regardless of the ST of the strain used [11]. It is worth mentioning that unlike the C57BL/6 mice used in this study, Ge *et al*. only specify using specific pathogen free-mice [11]. It must be presumed that these mice are BALB/c since this is the breed used for the immunization experiments conducted within the same publication [11]. C57BL/6 mice, which were used in the present study, are reliable for *S*. *suis* studies as they exhibit a prototypical Th1 immune response and a strong pro-inflammatory response [17, 44, 45]. On the other hand, BALB/c mice are the prototypical Th2 mouse breed [44]. As such, the innate immune response differs between these two breeds: C57BL/6 mice produce higher levels of the pro-inflammatory cytokine tumor necrosis factor (TNF) and the Th1 cytokine interleukin (IL)-12p70, in comparison to BALB/c mice [44, 46]. Moreover, macrophages isolated from C57BL/6 mice produce effector molecules required for bacterial killing, including nitric oxide, whereas those from BALB/c do not, resulting in impaired bactericidal activity of the latter [44].

In addition, the route of infection may also differentially affect the conclusions. Herein, bacteria were inoculated via the intraperitoneal route (IP), while the route of inoculation used by Ge *et al*. although not clearly stated, was probably intravenous (IV) [11]. Though bacteria will reach the bloodstream following IP inoculation via lymphatic drainage, the initial cell types activated will differ: IP inoculation results in activation of peritoneal macrophages while IV injection leads to immediate stimulation of blood leukocytes [47]. Indeed, it was previously reported that the route of infection had an effect on disease development following Group B *Streptococcus* infection [48, 49]. To our knowledge, the IV route of inoculation for *S*. *suis* in mice has been used in only a limited number of studies [50–52], while most mouse studies have used the IP route of infection for *S*. *suis*, as reviewed by Segura *et al*. [9].

Meanwhile, the role of the autolysin of a ST378 strain in virulence was previously evaluated using the zebrafish model of infection [13], in which the autolysin-deficient mutant presented attenuated virulence. However, using the C57BL/6 mouse model of infection, results from the present study indicate that the autolysin does not critically contribute to virulence and does not participate in host death, independently of the ST of the strain tested. An important difference between these studies, alongside the ST of the strains used, is the experimental design and the use of animal model. Though zebrafish possess innate and adaptive immune responses [53], the genetic differences with pigs and humans are greater than those between mice and pigs or humans [54]. Although ethical regulations facilitate the use of zebrafish over mice, the former are cold-blooded, are a model in which it is more difficult to conduct central nervous system studies (meningitis being the most important pathology caused by *S*. *suis* serotype 2), and are limited to lethal dose 50 studies [9]. Consequently, results obtained with zebrafish are difficult to extrapolate, which may limit their use in determining *S*. *suis* virulence factors.

Surprisingly, autolysin-deficiency resulted in increased virulence of the ST25 strain. This was unexpected since autolysin-deficient mutants of other pathogenic Gram-positive bacteria, including for LytA of *Streptococcus pneumoniae* [55] and AtlE of *Staphylococcus epidermidis* [56], were less virulent than their respective wild-type strains. Indeed, the *S*. *pneumoniae* autolysin, involved in cell wall remodeling, is responsible for the release of the pneumolysin, an important virulence factor of this pathogen [57]. Consequently, in the absence of the autolysin, it is possible that cell wall remodeling and protein secretion could be altered or halted, resulting in alteration of the ST25 bacterial strain surface architecture. It is well known that certain surface proteins of *S*. *suis* are important activators of the host cells [6, 8], and these may, by remaining attached to the bacteria or by being differentially expressed, contribute to inflammation and host death. Moreover, absence of the autolysin resulted in increased survival of the ST25 strain in blood, suggesting that this protein could play additional functions alongside those previously described for *S*. *suis* [13] as well as for other pathogenic streptococci [57]. Indeed, the *Staphylococcus saprophyticus* autolysin, Aas, was shown to bind sheep erythrocytes [58], although this function has not been described for *S*. *suis* so far. Further investigations will be required in order to better understand these differences in virulence and the possibly unique roles of this protein in ST25 strains.

## Conclusions

This study reiterates the urgent need in arriving to a consensus regarding the definition of *S*. *suis* serotype 2 virulence factors. Inconsistencies abound in the literature due to differences obtained between laboratories, and these have created a controversy that has only just been highlighted. The main source of these differences are variations and discrepancies in experimental design, including *in vitro* assays, cell lines, and animal models, which greatly affect the results, as demonstrated in this study for both the DPPIV and autolysin. Moreover, the use herein of different strain backgrounds has demonstrated that differences in bacterial characteristics and functions, alongside the role attributed to a virulence factor, may vary according to the *S*. *suis* serotype 2 strain. Consequently, it will be important to establish standard experimental designs, including methodology and appropriate cell lines and animal models, according to the experiment and purpose in order to facilitate comparison between laboratories. Alongside, studies should include strains of diverse origins in order to prevent erroneous and biased conclusions that could affect future studies. Finally, the use of alternative animal models cannot definitively exclude the role of a given *S*. *suis* virulence factor that may significantly contribute to disease during a natural infection in pigs. For example, the DPPIV has been reported to contribute to tissue degradation and perturbation of the host defense system [59], roles that although not critical in themselves, could significantly contribute to the final outcome of the natural infection by *S*. *suis*.

## Supporting information

S1 Appendix Evaluation of clinical signs and scoring following intraperitoneal injection of *Streptococcus suis* serotype 2 in mice. (PDF)
